# Simulating nitrogen management impacts on maize production in the U.S. Midwest

**DOI:** 10.1371/journal.pone.0201825

**Published:** 2018-10-22

**Authors:** Kamaljit Banger, Emerson D. Nafziger, Junming Wang, Umar Muhammad, Cameron M. Pittelkow

**Affiliations:** 1 Department of Crop Sciences, University of Illinois at Urbana-Champaign, Urbana, Illinois, United States of America; 2 Climate and Atmospheric Sciences Section, Illinois State Water Survey, Prairie Research Institute, University of Illinois at Urbana-Champaign, Urbana, Illinois, United States of America; Wageningen University, NETHERLANDS

## Abstract

Nutrient loss reduction strategies have recently been developed in the U.S. Midwest to decrease the environmental footprint associated with nitrogen (N) fertilizer use. Although these strategies generally suggest decreasing N rates and shifting the timing of N application from fall to spring, the spatiotemporal impacts of these practices on maize yield and fertilizer N use efficiency (NUE, kg grain yield increase per kg N applied) have not been assessed at the watershed scale using crop simulation models. We simulated the effects of N fertilizer rate (0, 168, 190, 224 kg N ha^-1^) and application timing [fall-applied N (FN): 100% N applied on 1 December; spring-applied N (SN): 100% N applied 10 days before planting; split N: 66% N applied on 1 December + 34% N applied 10 days before planting] on maize grain yield (GY) across 3042 points in Illinois during 2011–2015 using the DSSAT-CERES-Maize model. When simulations were scaled up to the watershed level, results suggest that increases in average maize GY for SN compared to FN occurred in years with higher than average winter rainfall (2011, 2013), whereas yields were similar (+/- 4%) in 2012, 2014, and 2015. Accordingly, differences in NUE for SN compared to FN were small (0.0–1.4 kg GY/kg N) when cumulative winter rainfall was < 300 mm, but increased to 0.1–9.2 kg GY/kg N when winter rainfall was > 500 mm at both 168 kg N ha^-1^ and 224 kg N ha^-1^. The combined practice of reducing N fertilizer amounts from 224 kg N ha^-1^ to 190 kg N ha^-1^ and shifting from FN to SN resulted in a wide range of yield responses during 2011–2015, with the probability of increasing yields varying from <10% to >70% of simulation points within a watershed. Positive impacts on both GY and NUE occurred in only 60% of simulations for this scenario, highlighting the challenge of simultaneously improving yield and NUE with a 15% N rate reduction in this region.

## Introduction

Maize grain yield (GY) has increased significantly in the past several decades, in part due to more efficient nitrogen (N) fertilizer use in high-yielding varieties [1–3]. In Illinois, maize occupies approximately 5.0 million ha (30% of total geographical area), and as a crop receives large amounts of N fertilizer [4]. Complex interactions among weather patterns, soil properties, crop growth, and N loss pathways make it difficult to synchronize fertilizer management with crop N demand, which may result in under- or over-N application [5]. Excessive N losses contribute to climate warming due to the potential for increased soil nitrous oxide emissions [6, 7], while also causing nutrient pollution of water resources [8, 9]. At a broader scale, maize production systems in Illinois are located within the Mississippi River Basin and contribute approximately 20% of nitrate loading to the Gulf of Mexico [10]. Hence, designing N management practices which increase maize GY while minimizing negative environmental consequences is crucial for this region.

In Illinois, the majority of N fertilizer is applied either late in the fall (fall-applied N, FN) in the form of ammonia—usually with nitrification inhibitor—or early in the spring, often as ammonia, before or near the time of planting (spring-applied N, SN). The uncertainty of wet spring weather, which has the potential to delay maize planting and other field operations, is an important reason that farmers perform land preparation activities such as N fertilizer application the previous fall. In addition, fall N application favors management logistics such as fertilizer availability, price, and workload distribution [11, 12]. The practice of applying N fertilizer 5–6 months before crop establishment and the period of active crop N uptake begins, however, increases the potential for N losses through leaching or denitrification [13, 14]. Despite FN being a common practice in this region, a consensus has not been reached regarding the effectiveness of N fertilizer application timing on crop yield, nitrogen use efficiency (NUE), and environmental sustainability [15–18].

Previous studies have suggested that weather early in the growing season is the most important factor determining the effects of FN compared to SN on crop yield. In the U.S. Midwest, Bundy [14] concluded that FN is 10–15% less effective than SN for maize cropping systems. In contrast, Boswell et al. [18] did not observe differences in maize GY among FN, SN, and split N applications consisting of fall and spring N portions in the southeastern U.S. In a three-year field experiment, Vetsch and Randall [19] found that maize GY was 2 Mg ha^-1^ lower with FN than SN in 1999, but was not different in 1997 and 1998. They concluded that an extremely wet early spring in 1999 (66 mm more rainfall than the long-term mean) likely increased leaching and/or denitrification losses, which reduced crop uptake for FN compared to SN, whereas crop uptake and yields were similar in years with normal precipitation. On the other hand, in years with limited precipitation dry soils can inhibit the transport of inorganic N to plant roots, resulting in lower N losses and similar N recovery efficiency under different fertilizer application timings [20]. In addition to precipitation, N rate is an important factor for predicting the impacts of SN vs FN. Over a 5-year field experiment in Minnesota, maize GY was 14% greater in SN (9.4 Mg ha^-1^) than FN (8.2 Mg ha^-1^) at 134 kg N ha^-1^ [21], yet increasing the fertilizer amount to 202 kg N ha^-1^ decreased the differences in maize GY to 5% between the SN (10.5 Mg ha^-1^) and FN (10.0 Mg ha^-1^). Given that fertilizer application timing impacts on GY are regulated by complex interactions among weather patterns, fertilizer rate, and soil properties, a more quantitative understanding of conditions under which SN is likely to result in higher yields and NUE is needed.

Recently, results generated from field trials have been upscaled to the watershed level to aid in the formulation of strategies for reducing nutrient pollution in waterbodies. For example, it was estimated in the Minnesota Nutrient Reduction Strategy that delaying N fertilizer application from FN to SN would also reduce optimum N rates, with the combined impact of these two practices lowering N leaching losses by 26% in tile drained fields [22]. In contrast, in the Iowa Nutrient Reduction Strategy, the decrease in N leaching for SN compared to FN was estimated to be 6±25% based on field results and assumptions about changes in optimum N rates [23]. In Illinois, federal and state agencies have set an ultimate goal to reduce total nitrate loads in rivers by 45% compared to 1980–1996 baseline levels, with an intermediate milestone of 15% reduction by 2025 [10]. While various in-field and edge-of-field strategies are promoted for reducing nitrate losses in Illinois, one reduction scenario is based on shifting N application timing from FN to SN, with a corresponding 15–20% reduction in nitrate losses [10].

The importance of conducting N management field trials is fundamental for improving nutrient loss reduction strategies. In Illinois, approximately 83 N rate experiments were carried out in 2014–2015 to improve the maximum return to N (MRTN) rate recommendations for maize production [24]. However, there are inherent factors limiting the quantity of field experiments that can be conducted under different soil types and climate conditions in this region, including economic and time constraints. Thus, crop simulation models represent a complementary approach to further investigate the potential impacts of recommended N management practices in state-level nutrient loss reductions strategies on maize GY and NUE. Crop models are increasingly used as a tool to explore the spatiotemporal impacts of different management scenarios following calibrations at field experiments, particularly for upscaling the impacts of N management from field to watershed and regional scales [25–28]. Despite agricultural nutrient losses representing a serious challenge facing farmers, environmental scientists, and policy makers in the U.S. Midwest, few studies have modeled the potential outcomes of changes in N management on maize GY at the regional scale.

The objective of this study was to evaluate the impacts of N fertilizer amount and timing on maize GY and NUE in Illinois using the Decision Support System for Agrotechnology Transfer (DSSAT) CERES-Maize model. First, the model was calibrated with six field experiments that had N fertilizer rate and timing treatments during 2015–2017. Second, the performance of the model was validated using maize GY data from 15 independent field experiments. Third, the calibrated model was applied at 3042 points in Illinois during 2011–2015 to assess i) changes in simulated yield due to N fertilizer amount and timing, ii) the impacts of winter rainfall on NUE, ii) opportunities for simultaneously increasing GY and NUE, and iii) the probability of maize GY increasing when switching from FN to SN and reducing N rates by 15%.

## Materials and methods

### DSSAT-CERES-Maize model

DSSAT (version 4.5) simulates daily crop growth and development [29, 30] and has been used to estimate crop productivity in response to various agronomic management and climate scenarios as well as to optimize nutrient use in crop production systems [31–34]. Modular structure of DSSAT includes weather, soil, soil-plant-atmosphere interface, and management modules which drive the cropping system model plant growth module. Detailed information on the background and functions of DSSAT can be obtained in previous publications [30, 35–44].

In brief, the weather module reads or generates daily weather data, while the soil-plant-atmosphere module accounts for competition for light and water among the soil, plants, and atmosphere [30]. The soil module includes a soil water balance and soil N/organic matter decomposition sub-models. The soil water balance is simulated at a daily time step based on precipitation, irrigation, evapotranspiration, runoff, and drainage from the soil profile [45]. The soil water deficit is used as a parameter that reduces plant biomass production [45]. In the original DSSAT model, the soil organic matter (SOM) decomposition was based on the PAPRAN model, which has also been called the CERES-based SOM–residue module [46]. The PAPRAN model was primarily developed for annual pastures and small grain crops in semiarid environments, with soil organic matter pools including fresh organic matter and humidified soil organic matter. However, this model has been shown to produce significant uncertainties for predicting N release from soil organic matter decomposition [43]. Therefore, more recently the CENTURY module has been embedded into DSSAT to improve soil carbon and N simulations, with CENTURY distinguishing between active, slow, and passive soil organic matter pools [44]. DSSAT uses CERES-Maize to simulate crop growth and development based on physiological processes that describe the response of maize to soil and other environmental conditions [30, 42]. In this study, we estimated maize GY response to different N management treatments. Nitrogen use efficiency (NUE) for each N fertilizer treatment was calculated following Wang *et al*., [47]:

$$NUE=\frac{\left({GY}_{fert}–{GY}_{control}\right)}{N\;fertilizer\;applied}$$

where GY_fert_ is maize GY (kg ha^-1^) of the N fertilizer treatment, GY_control_ is the maize GY (kg ha^-1^) in the control treatment without N fertilizer, and N fertilizer applied (kg ha^-1^) is the N rate for that treatment.

### Input datasets

Model simulations required the use of soil information, daily weather parameters, soil initial conditions, and crop management information [30]. Soil parameters were directly extracted from the Gridded Soil Survey Geographic data (gSSURGO) [48], while drained upper and lower limit, saturation, drainage coefficients, and runoff curve number were estimated based on soil profile properties [30]. Weather parameters were obtained from 18 weather stations in the Illinois Climate Network (http://www.isws.illinois.edu/warm/datatype.asp), and were transformed into the DSSAT input format using the WeatherMan program [49].

Soil initial conditions (including soil ammonium and nitrate status) at each site were developed based on data from six field experiments in Illinois used for calibration purposes as described below (S1 Table). For crop management information, dry seed was planted at 4 cm depth in rows with a plant population at seeding of 8 plants m^-2^ for all simulations. Fertilizer source, amount, timing, and method of application were based on data from individual field trials used for model calibration and validation in this study.

### Model calibration

In the DSSAT calibration process, six genotype coefficients and one N stress factor for maize were improved using crop phenology stages and maize GY data from multiple field experiments in Illinois during 2014–2015 (S1 and S2 Tables). Six cultivar specific coefficients include parameters for controlling early growth (P1, P2 and P5), grain filling (G2 and G3), and phylochron interval between successive leaf tip appearances (PHINT). P1 (degree days above a base temperature of 8°C) is the thermal time from seedling emergence to the end of the juvenile phase during which the plant is not responsive to changes in photoperiod. P2 (number of days) is the extent to which development is delayed for each hour increase in photoperiod above the longest photoperiod at which development proceeds at a maximum rate (which is considered to be 12.5 h). P5 (degree days above a base temperature of 8°C) is the thermal time from silking to physiological maturity. G2 is the maximum possible number of kernels per plant; G3 (mg d^−1^) is the kernel filling rate during the linear grain filling stage under optimum conditions; and PHINT (degree days) is the phylochron interval, i.e., the interval in thermal time between successive leaf tip appearances.

A default maize cultivar, DeKalb 485, was selected in the model calibration. Early growth (P1, P2 and P5) parameters were improved using six N tracking experiments (S1 Table). These parameters were primarily associated with improving the crop phenological stages in the model. We used maize GY data from six N tracking and three N rate experiments to improve the maize GY related model parameters. In this process, genotype parameters associated with grain filling (G2 and G3) and the N stress factors were improved in the model (S2 Table). In the N tracking experiments, four field experiments had the following N treatments: 1) control, 2) 224-Fall (224 kg N ha^-1^ applied as anhydrous ammonia in fall), 3) 224-Spring (224 kg N ha^-1^ applied as anhydrous ammonia in spring), 4) 224-Split1 (56 kg N ha^-1^ applied as UAN at planting + 168 kg N ha^-1^ applied as UAN at V5 stage), 5) 224-Split2 (112 kg N ha^-1^ applied as anhydrous ammonia in fall + 56 kg N ha^-1^ applied as UAN at planting and again at V5 stage) (S1 Table). Two other N tracking experiments located in southern Illinois included the following N treatments: 1) control, 2) 168-Spring (168 kg N ha^-1^ applied as UAN at planting), 3) 168-Split1 (56 kg N ha^-1^ applied as UAN at planting + 112 kg N ha^-1^ applied as UAN at V5 stage), and 4) 168-Split2 (56 kg N ha^-1^ applied as UAN at planting + 112 kg N ha^-1^ applied as UAN at V9 stage). Three N rate experiments (S2 Table) included the following N treatments: 0 (control), 56, 112, 168, 224, and 280 kg N ha^-1^ (S2 Table).

We adjusted three genotype coefficients (P1, P2, and P5) to reduce the difference between anthesis and physiological maturity dates in the DSSAT model predictions and six N tracking experiments in 2015 (Table 1). The P1 and P5 parameter values were adjusted to 210 and 800, respectively. The calibrated parameters reduced the DSSAT predicted vs field observed difference in the anthesis and physiological maturity dates to ±3 days in six N tracking experimental sites. A combination of maize GY data from three N rate and six N tracking experimental site were used to calibrate the N stress factor and three genotype parameters (G2, G3, and PHINT). The G2 and G3 parameters were slightly increased to minimize the difference in the maize GY predicted by the DSSAT vs field observed data during 2014 and 2015 (Table 1). In addition to the cultivar parameters, we modified SOM decomposition parameters in the DSSAT model as part of another study focused on modeling inorganic soil N status [50]. Using the uncalibrated model, we found that crop termination occurred before reaching maturity due to low N availability in some cases when SOC was less than 1.0% and soils were well drained. This resulted in maize GY being very sensitive to N fertilizer additions when compared to field data from the three N experiments. Therefore, during calibration we adjusted N availability by improving the SOM decomposition parameters in the DSSAT module. Calibrated model parameters are provided in Table 2.

**Table 1:** Calibrated maize genotype and nitrogen stress factor of the DSSAT model.

Parameter	Default value	Calibrated value
P1	215	210
P2	0.60	0.60
P5	785	800
G2	750	900
G3	8.7	10.0
PHINT	45	50
FSLFN	0.05	0.50

P1: Thermal time from seedling emergence to the end of the juvenile phase during which the plant is not responsive to changes in photoperiod. P1 is expressed in degree days above a base temperature of 8° C.

P2: Extent to which development (days) is delayed for each hour increase in photoperiod above the longest photoperiod at which development proceeds at a maximum rate (which is considered to be 12.5 hours)

P5: Thermal time from silking to physiological maturity (expressed in degree days above a base temperature of 8° C).

G2: Maximum possible number of kernels per plant

G3: Kernel filling rate during the linear grain filling stage and under optimum conditions (mg/day)

PHINT: Phylochron interval; the interval in thermal time (degree days) between successive leaf tip appearances.

FSLFN: Fraction of leaf area senesced under 100% nitrogen stress, 1/day

**Table 2:** Calibrated SOM decomposition parameters of the DSSAT model.

Parameter	SLDR (≤0.05)		SLDR (0.25)		SLDR (0.40)		SLDR (≥ 0.60)	
	SOC (<1.0%)	SOC (≥1.0%)	SOC (<1.0%)	SOC (≥1.0%)	SOC (<1.0%)	SOC (≥1.0%)	SOC (<1.0%)	SOC (≥1.0%)
DECS1(0)	0.05644	0.05644	0.05644	0.05644	0.05644	0.05644	0.05644	0.05644
DECS1(1)	0.04000	0.02000	0.03000	0.03000	0.03000	0.03000	0.03000	0.03000
DECS2(1)	0.00084	0.000188	0.00048	0.00028	0.00058	0.00039	0.00058	0.00038
DECS3(1)	0.00008	0.000012	0.000112	0.000012	0.000087	0.000016	0.000082	0.000012
DECSTR(0)	0.01068	0.01068	0.01068	0.01068	0.01068	0.01068	0.01068	0.01068
DECSTR(1)	0.01342	0.01342	0.01342	0.01342	0.01342	0.01342	0.01342	0.01342

DECS 1(0), DECS1 (1), DECS2 (1), DECS3 (1), and DECSTR are the decomposition rates of different Soil Organic Matter Pools.

### Model evaluation

**Fig 1:**
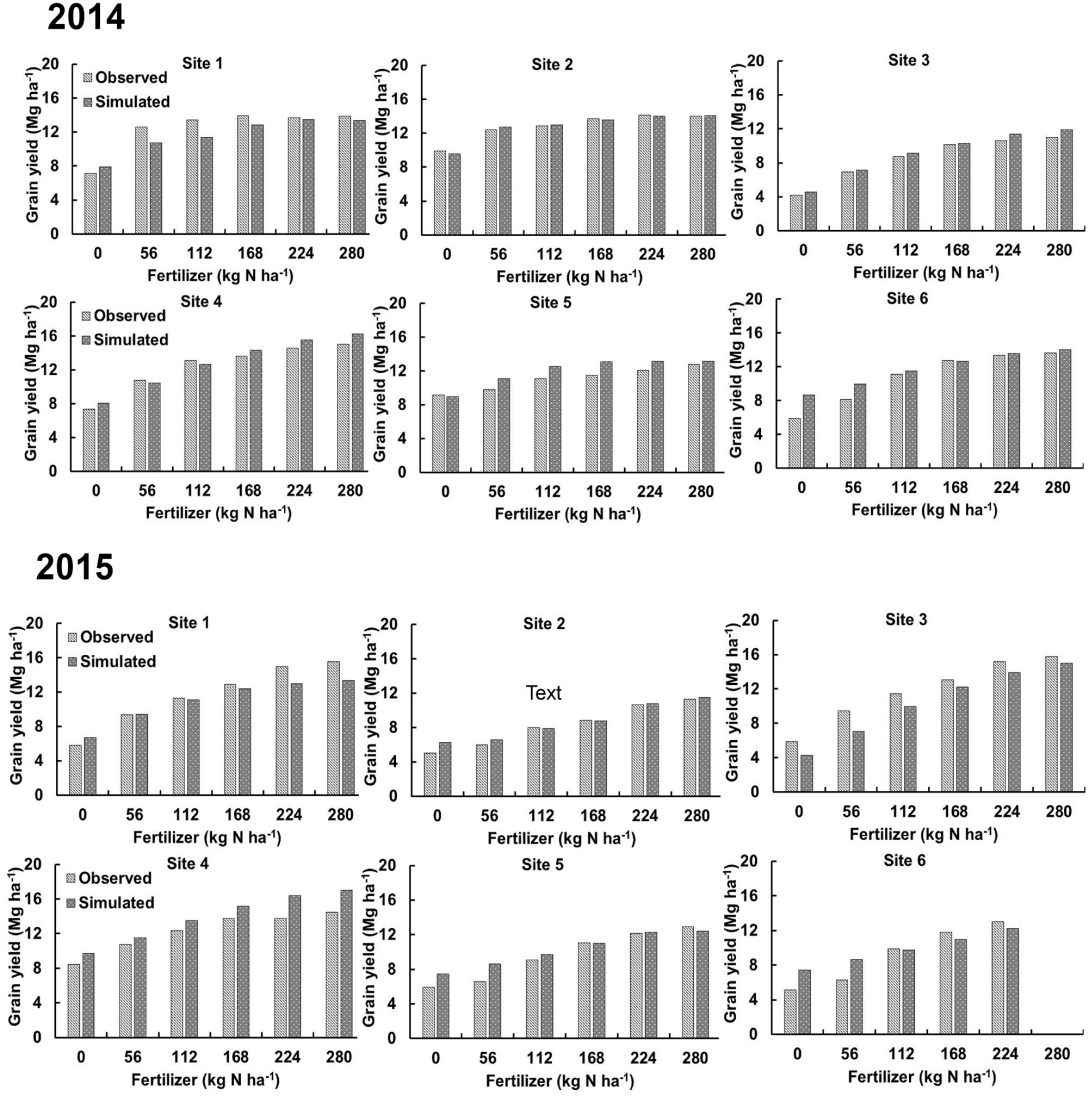
Comparison of observed and DSSAT simulated maize grain yield according to N fertilizer amount. Simulations were performed for different N fertilizer amounts at six sites across two years in Illinois.

**Fig 2:**
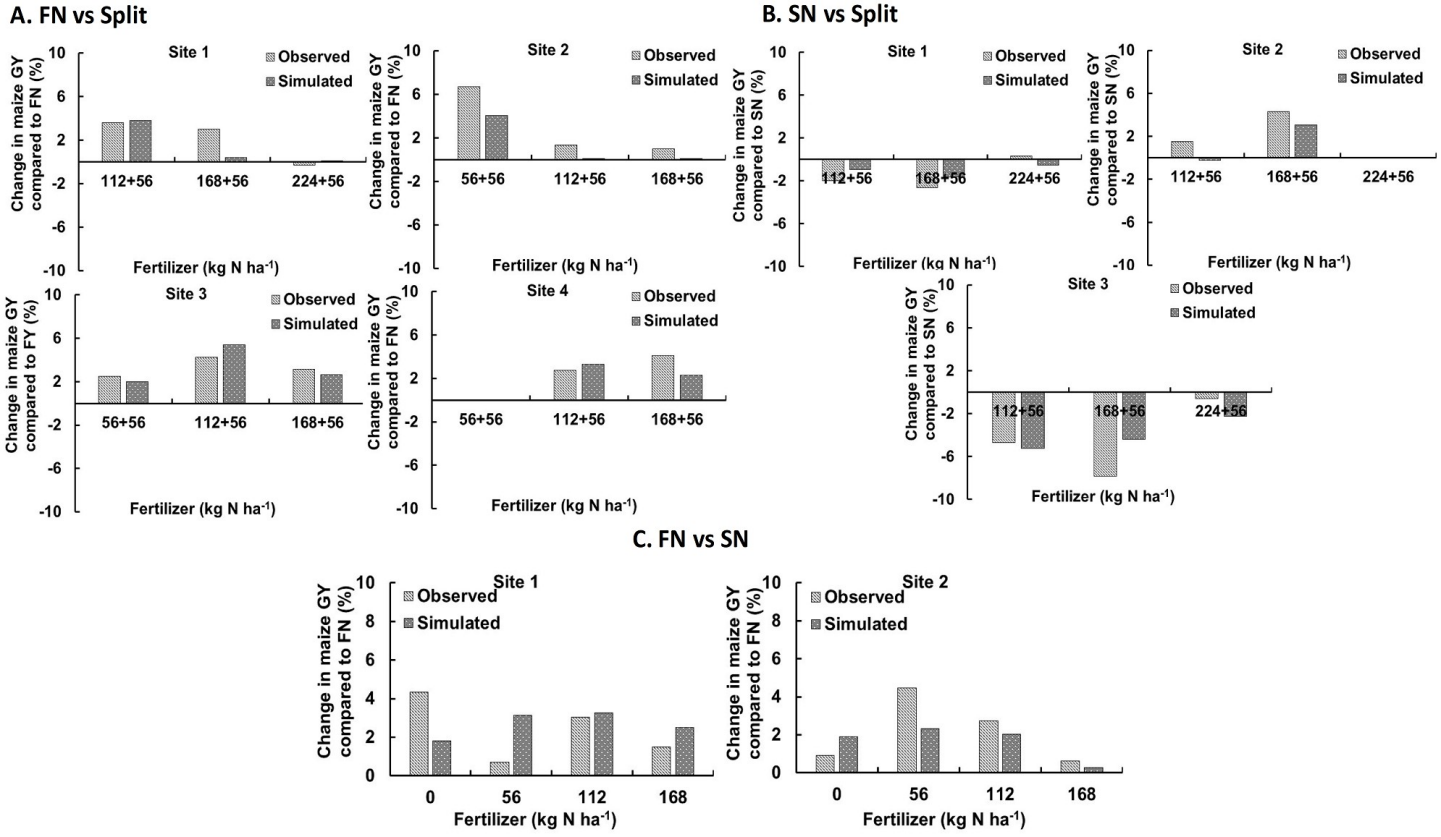
Comparison of observed and DSSAT simulated maize grain yield according to N fertilizer application timing. A) fall applied N with equal amounts of N applied in two splits, B) spring applied N with equal amounts of N applied in two splits, and C) fall with spring applied N in different field experiments in 2015.

The performance of model simulations in predicting the effects of N fertilizer amount and timing on maize GY was validated using 15 independent field experiments (Figs 1 and 2), which were located in different soil types (S3 Table). To determine model performance, we calculated the following four indicators: coefficient of determination (r^2^), normalized Root Mean Square Error (nRMSE), coefficient of residual mass (CRM), and index of agreement (D-index). Normalized RMSE quantifies the relative difference between model simulated (E*i*) and observed (M*i*) data. In general, model performance is considered as ‘excellent’ when nRMSE is less than 10%, ‘good’ when nRMSE is greater than 10% but less than 20%, ‘fair’ when nRMSE is greater than 20% but less than 30%, and ‘poor’ when nRMSE is greater than 30% [31, 51].

$$nRMSE=\sqrt{\sum \frac{{\left(Ei-Mi\right)}^{2}}{n}\times \frac{100}{\overset{-}{M}}}$$

The CRM index is used to determine if the model is over- or underestimating the observed data [31, 52]. A negative CRM value indicates a tendency of the model to overestimate, while a positive CRM value indicates a tendency of the model to underestimate the observed data.

$$CRM=1-\frac{\sum_{i=n}^{n}Ei}{\sum_{i=1}^{n}Mi}$$

The D-index reflects the degree to which the observed variation is accurately estimated by the simulated variation. D value ranges from 0 to 1 [53]. D-index values closer to 0.0 indicate no agreement, while the D-value of 1 indicates that model perfectly captures the dispersion in the data [53]. In general, D-index value of less than 0.50 suggest greater diversity and inconsistency in model predictions compared to simulated values [31].

$$D-index=1-\frac{\sum_{i=1}^{n}{\left(Mi-Ei\right)}^{2}}{\sum_{i=1}^{n}{\left(|Ei-\overset{-}{M}|+|Mi-\overset{-}{M}|\right)}^{2}}$$

In the model validation process, data from six independent field experiments consisting of large N rate strips were used to determine the impact of N fertilizer amount (0, 56, 112, 168, 224, and 280 kg N ha^−1^) on maize GY during 2014 and 2015. The following crop management information was used at each site: planting method (dry seed), planting distribution (rows), plant population at seeding (8 plants m^-2^), planting depth (4 cm), and drainage type (tile drainage). The field experiments were located in different geographical regions within Illinois, and maize GY ranged from <5.0 Mg ha^-1^ to > 16.0 Mg ha^-1^ during 2014–2015 (Fig 1). The calibrated DSSAT model was able to predict maize GY in different N fertilizer treatments with a mean deviation of 0.85 Mg ha^-1^ from field observed values. To evaluate DSSAT model performance in predicting the impact of fertilizer application timing, we used nine field experiments that had equal amounts of N fertilizer applied in different times (Fig 2). Of these nine experiments, four had FN vs fall + 56 kg N ha^-1^ applied in spring, three had early spring vs early spring + 56 kg N ha^-1^ applied late in the spring, and two had FN vs SN comparisons under several fertilizer application amounts. The DSSAT model accurately predicted that fall + 56 kg N ha^-1^ applied in spring always had higher maize GY than equal amounts of N applied in a single dose in the fall (Fig 2a). However, splitting spring application (SN vs Split) reduced maize GY in two of the three experimental sites (Fig 2b). In the two field experiments having FN vs SN comparisons, DSSAT predictions were closer to field observed data with GY being 1–4.5% higher in SN than FN (Fig 2c).

**Fig 3:**
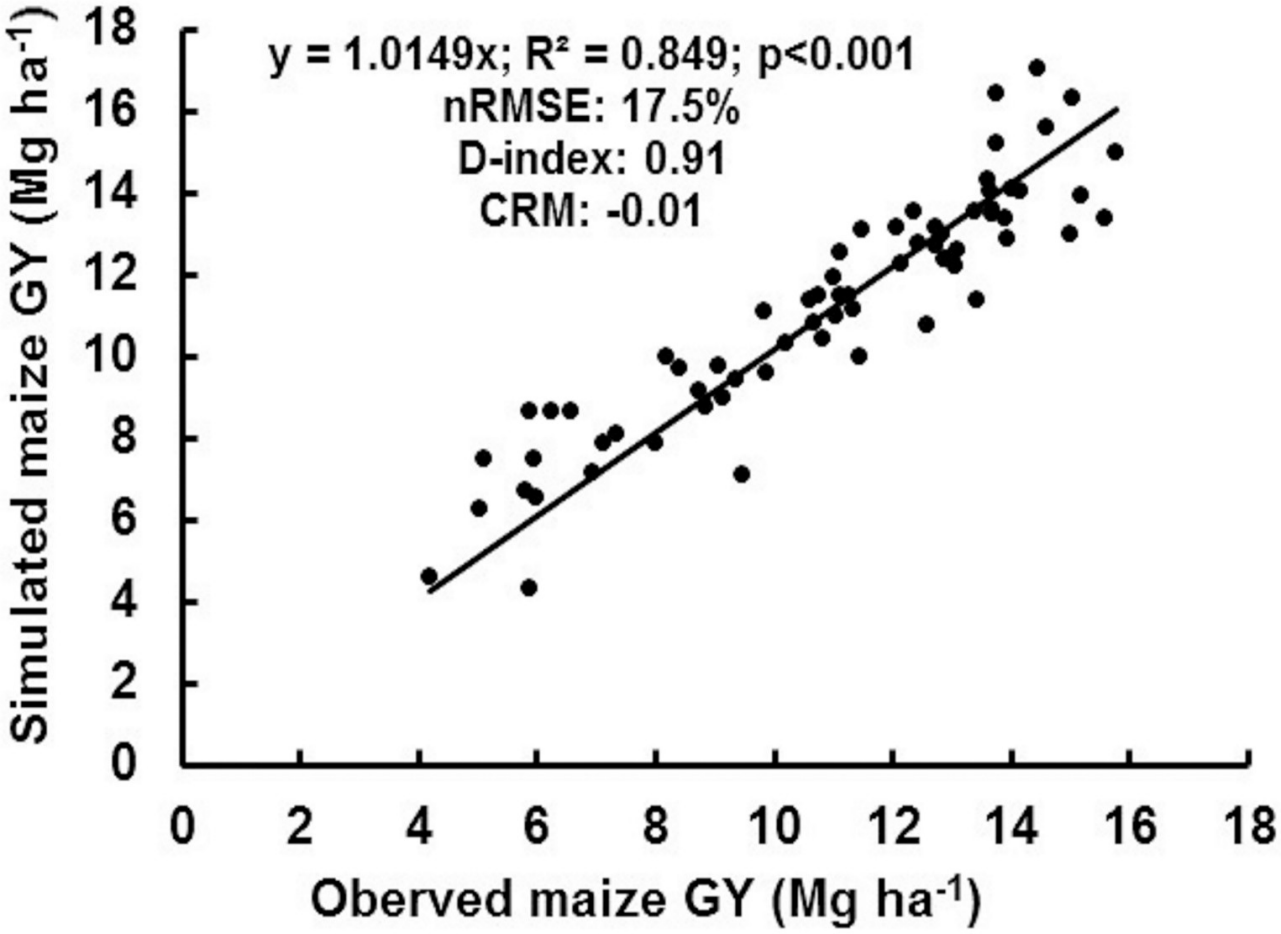
Model performance criteria for assessing the ability of DSSAT to predict maize grain yield. Simulations include experiments for N fertilizer amount and timing treatments.

Overall, based on nRMSE (17.5%) and D-index (0.91) values, model performance was considered “good” in predicting maize GY across N fertilizer amounts and timing treatments in Illinois (Fig 3). The CRM index value of –0.01 suggests the model slightly overestimated maize GY as compared to field observed values.

### Model automation and upscaling

A program was developed in MATLAB to automate the DSSAT simulations for a total of 3042 points across Illinois (S1 Fig). To generate these simulation points, we used cropland area as a mask to generate 3042 data points at a 5 km by 5 km grid over Illinois. For running the DSSAT model at each geographical location, the automation program generated the weather and soils data files based on a proximate weather station and the gSSURGO dataset, respectively [48]. Soil initial conditions at each site were based on the data obtained from six Illinois field experiments including soil ammonium and nitrate status (S1 Table).

Average maize planting dates for each region were obtained from weekly USDA National Agricultural Statistics Service’s Illinois crop progress and condition reports (https://www.nass.usda.gov/Statistics_by_State/Illinois/Publications/Crop_Progress_&_Condition/). At each site, maize planting date was set to the date when 50% of the geographical area in a crop district had been planted (planting dates ranged from 17 April to 23 May during 2011–2015). In this way, maize planting dates for all simulation points within a crop district were the same each year (S2 Fig). Crop management practices including sowing method, plant population density at seeding, planting depth, drainage, and fertilizer application method were consistent across all simulations. Six N fertilizer application scenarios were included: 1) control (0 kg N ha^-1^), 2) 168 FN (168 kg N ha^-1^ applied as anhydrous ammonia on 1 December), 3) 168 SN (168 kg N ha^-1^ applied as UAN 10 days before planting), 4) 224 FN (224 kg N ha^-1^ applied as anhydrous ammonia on 1 December), 5) 224 SN (224 kg N ha^-1^ applied as UAN 10 days before planting), and 6) 112 FN+56 SN (112 kg N ha^-1^ applied as anhydrous ammonia on 1 December + 56 kg N ha^-1^ applied as UAN at planting). As such, FN occurred in December of the year prior to maize planting, while SN occurred in April or May of the maize planting year.

From the 3042 point simulations for each treatment each year, values falling between the 5^th^ and 95^th^ percentiles were upscaled to 56 hydrological unit codes-8 (HUC-8) using the average of data points in each watershed. Based on the watershed delineations, the U.S. is divided HUC at four levels: regions, sub-regions, accounting units, and cataloging units, which are nested within each other from the largest geographic area (regions) to the smallest geographic area (cataloging units). A hydrologic unit is the land area that drains into hydrologic feature, such as a stream, river, or lake. In general, hydrological units are synonymous with watersheds when their boundaries include all the source area contributing surface water to a single defined outlet. However, the hydrologic unit boundaries do not always surround a complete watershed but may delineate truncated portions of a larger watershed. In this way, a single or a combination of multiple hydrological units may form a single watershed. The HUC-8 represents the hydrological units at cataloging units levels which the INLRS estimates and prioritize watersheds based on N losses from hydrological unit codes-8 [24]. In the current study, 16 HUC-8 in the southern Illinois were excluded since FN is not applied in southern Illinois due to concerns of greater N losses associated with warmer soil temperatures in southern Illinois [54].

To determine if a quantitative relationship exists between cumulative early spring rainfall and soil drainage conditions on the effectiveness of N fertilizer timing, we categorized results for NUE across four rainfall gradients (< 300, 300–400, 400–500, and >500 mm) and four drainage classes (very poorly drained, somewhat poorly drained, moderately well drained, well and extremely drained soils). The probability of yields increasing due to the combined practice of reducing N fertilizer amount by 15% (224 to 190 kg N ha^-1^) and delaying application time from fall to spring was also estimated at the watershed scale during 2011–2015. This scenario represents a conservative reduction in N rate based on moving from the high end to the low end of the profitable N rate range surrounding the MRTN rate developed for Illinois. Probability of yield increase was calculated as the percentage of simulations with yield increases greater than 100 kg ha^-1^ within each watershed using ArcGIS software. Likewise, yield decreases resulting from this change in N management were defined as a risk factor. The risk factor for switching from 224 FN to 190 SN was calculated as:

Riskfactorforyieldpenalty=100-probabilityofyieldincrease

## Results

### Rainfall patterns during 2011–2015

**Fig 4:**
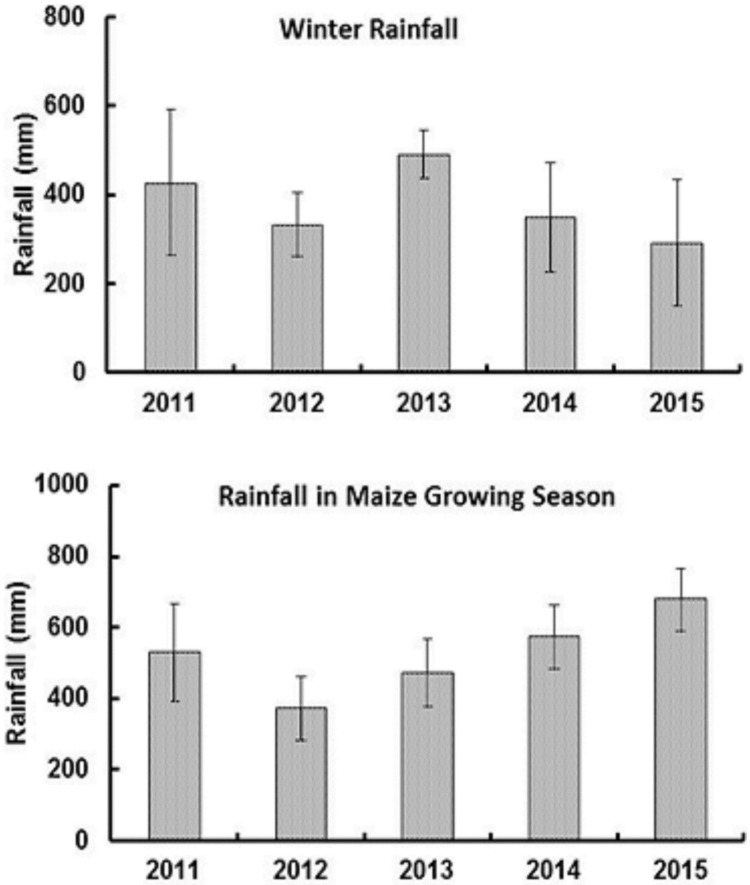
Rainfall in winter (December-April) and maize growing season (May-September) rainfall across 18 Illinois weather stations.

Across 18 weather stations in Illinois, annual rainfall ranged from 703 mm to 969 mm during 2011–2015, which showed significant variation within and across years (Fig 4). Cumulative winter rainfall (December through April) across the 18 weather stations varied two-fold, ranging from 291±143 mm to 490±53 mm during 2011–2015. Among the years, 2011 and 2013 received 78–200 mm greater winter rainfall (427–490 mm) compared with other years (291–349 mm). On the other hand, 2015 had the wettest growing season with cumulative rainfall (May through September) being 100–300 mm greater (677±88 mm) than the other years (370–572 mm). In contrast, 2012 received 21–45% lower rainfall in the maize growing season (370±89 mm) compared to the other years (472–677 mm), resulting from widespread drought in 2012 [55].

### Impact of N fertilizer amount and timing on maize grain yield

**Fig 5:**
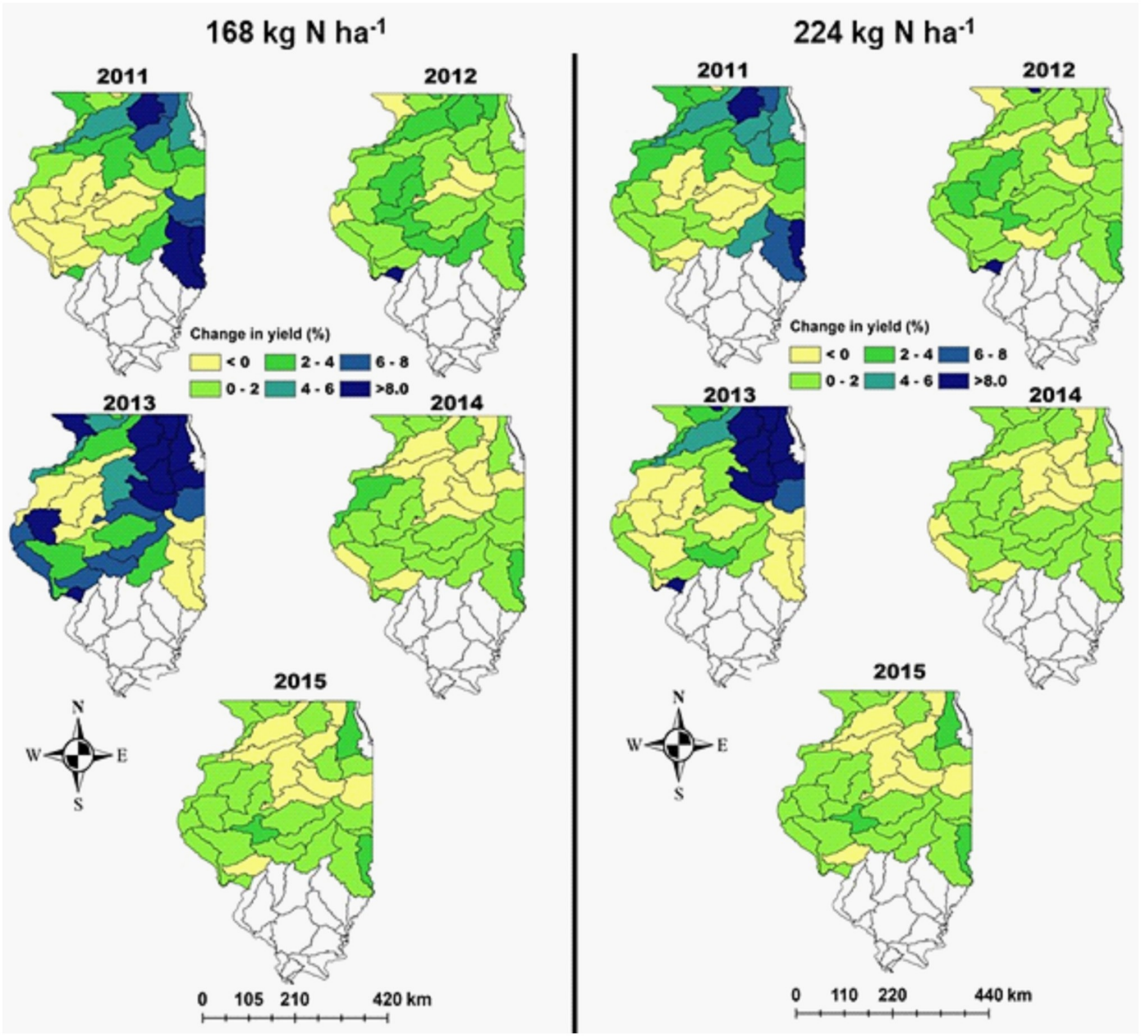
Change in average DSSAT simulated maize grain yield at the watershed scale. Points in each watershed were averaged for spring (10 days before planting) compared with fall fertilizer N application (1 December) at two N rates (168 and 224 kg N ha^-1^) during 2011–2015. Southern Illinois watersheds have been excluded as FN is not applied in southern Illinois.

When results across all simulations in a watershed were averaged, shifting fertilizer application timing from FN to SN for 168 kg N ha^-1^ increased maize GY by more than 2% in 16, 11, 31, 2, and 3 of 40 watersheds during 2011, 2012, 2013, 2014, and 2015 (Fig 5). Average maize GY otherwise remained within +/- 2% for FN and SN for the remaining watersheds each year. The mean yield increase with SN was highly variable across years and different regions of Illinois, ranging from 1–19% and 0–18% at 168 kg N ha^-1^ and 224 kg N ha^-1^, respectively. In 2013 maize GY differences > 8% for SN compared to FN occurred in 17 watersheds at 168 kg N ha^-1^, but only 10 watersheds at 224 kg N ha^-1^. In 2011, maize GY for SN was > 8% greater than FN in 7 and 4 of 40 watersheds at 168 kg N ha^-1^ and 224 kg N ha^-1^, respectively. In contrast, for 168 kg N ha^-1^ maize GY for SN was within ±4% of FN for all watersheds except 1 in 2012, and all watersheds in 2014 and 2015.

**Fig 6:**
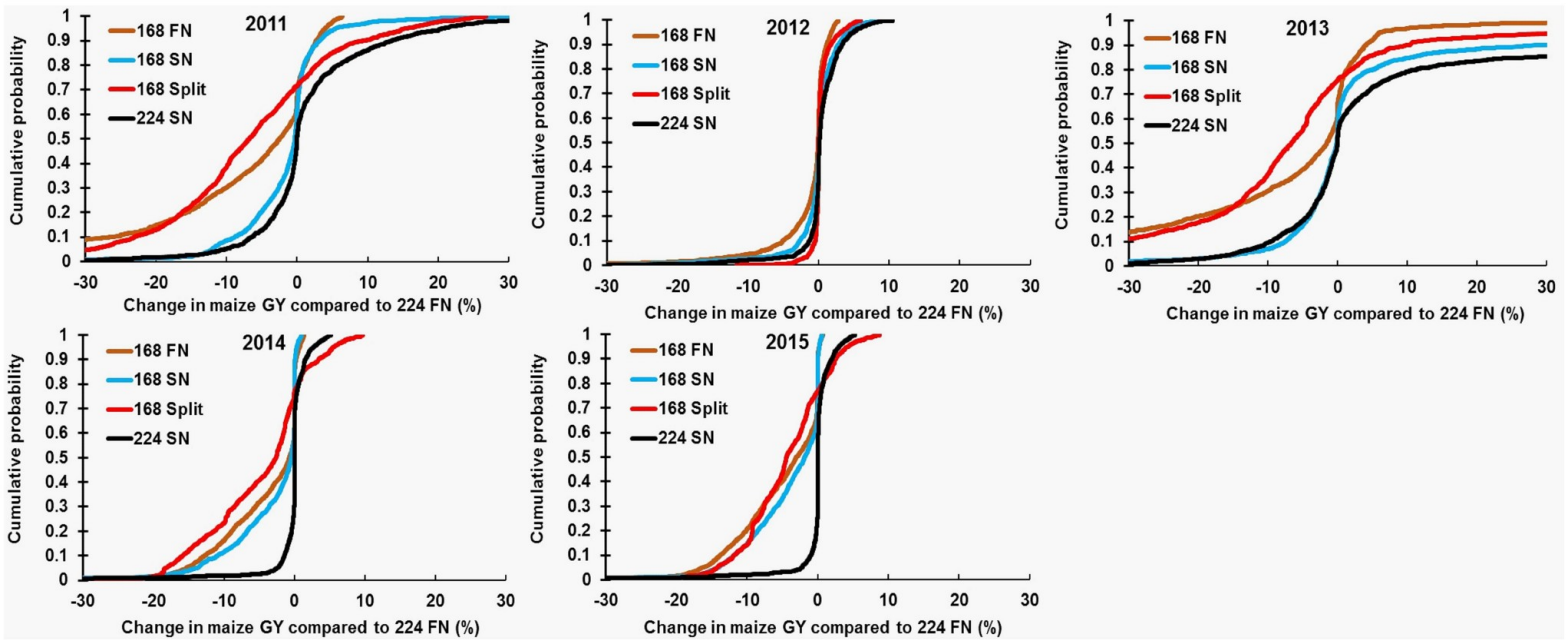
Cumulative probability distributions for change in maize grain yield. Individual fertilizer treatments were compared to 224 FN (224 kg N ha^-1^ applied in fall) within each site during 2011–2015. Control (0 kg N ha^-1^); 224 FN (224 kg N ha^-1^ applied on fall); 224 SN (224 kg N ha^-1^ applied spring); 168 FN (168 kg N ha^-1^ applied in fall); 168 SN (168 kg N ha^-1^ applied in spring); 168 Split (112 kg N ha^-1^ applied in fall + 56 kg N ha^-1^ applied in spring). Southern Illinois watersheds have been excluded as FN is not applied in southern Illinois.

Cumulative probability distributions showing the difference in maize GY of N management alternatives compared to 224 FN within each site are presented in Fig 6. Compared to 224 FN, 50% of simulations (25^th^ and 75^th^ percentiles) fell within the range of –7 to 4% difference in maize GY for 168 FN, 168 SN, and 168 Split during 2011–2015 (Fig 6). The greatest reduction in median maize GY for 168 FN and 168 SN occurred in 2015, whereas 168 Split was lowest in 2013. In contrast, maize GY in 2012 varied within ±1.9% of 224 FN for all treatments (except 168 Split) in 50% of simulations. When comparing 224 SN to 224 FN, 50% of simulations had maize GY within an average range of –1.1 to 3.3% during 2011–2015 (medians ranged from 0–0.2%). For the 20% of simulations where 224 SN increased GY compared to 224 FN, roughly half of the cases occurred in 2013 (average yield increase of 11%), with this year receiving 40–68% higher rainfall in winter compared to other years. Across the remaining years, approximately 10% of simulations resulted in >3% yield increase.

### Impact of N fertilizer amount and timing on NUE

**Fig 7:**
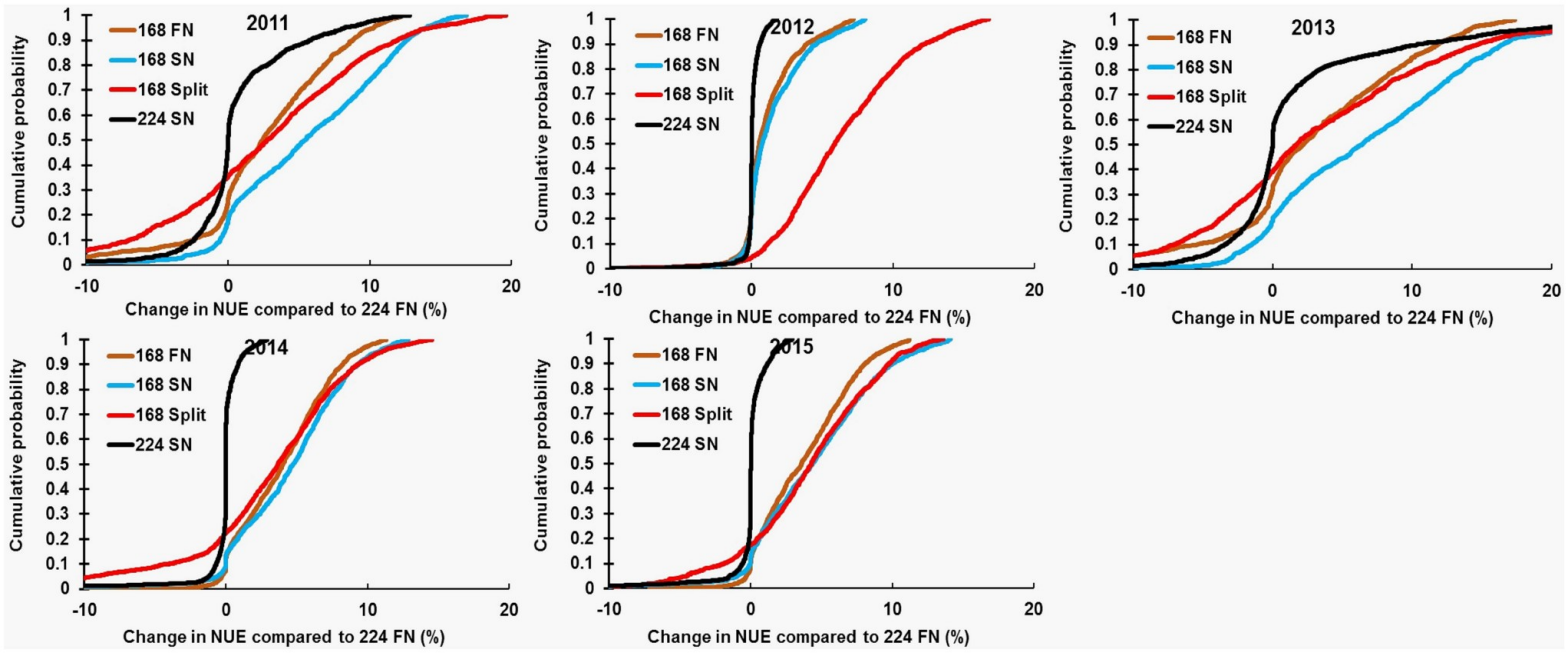
Cumulative probability distributions for change in N use efficiency. Individual fertilizer treatments were compared to 224 FN (224 kg N ha^-1^ applied in fall) within each site during 2011–2015. Control (0 kg N ha^-1^); 224 FN (224 kg N ha^-1^ applied on fall); 224 SN (224 kg N ha^-1^ applied spring); 168 FN (168 kg N ha^-1^ applied in fall); 168 SN (168 kg N ha^-1^ applied in spring); 168 Split (112 kg N ha^-1^ applied in fall + 56 kg N ha^-1^ applied in spring). Southern Illinois watersheds have been excluded as FN is not applied in southern Illinois.

During 2011–2015, the median difference in NUE of N management alternatives compared to 224 FN within each site ranged from 0 to 8.6 kg GY/kg N (Fig 7). In 2012, the difference in NUE at 50% cumulative probability was lower than other years (with the exception of 168 Split), since a drought reduced crop growth and development in 2012. Across years, the median difference in NUE compared to 224 FN within each site for the three treatments receiving 168 kg N ha^-1^ (median: 0.7–8.5 kg GY/kg N for 168 FN, 168 SN, and 168 Split) was greater compared to 224 SN (median: 0 to 2.2 kg GY/kg N). Differences in NUE between SN and FN at 224 kg N ha^−1^ were relatively minor (averaging 0.7 kg GY/kg N), whereas 168 SN further increased the NUE difference for 168 FN compared to 224 FN by an average of 2.0 kg GY/kg N. Among years, median NUE differences between 224 SN and 224 FN were greater in 2011 and 2013 (1.3–2.2 kg GY/kg N) than other years (0–0.2 kg GY/kg N).

**Fig 8:**
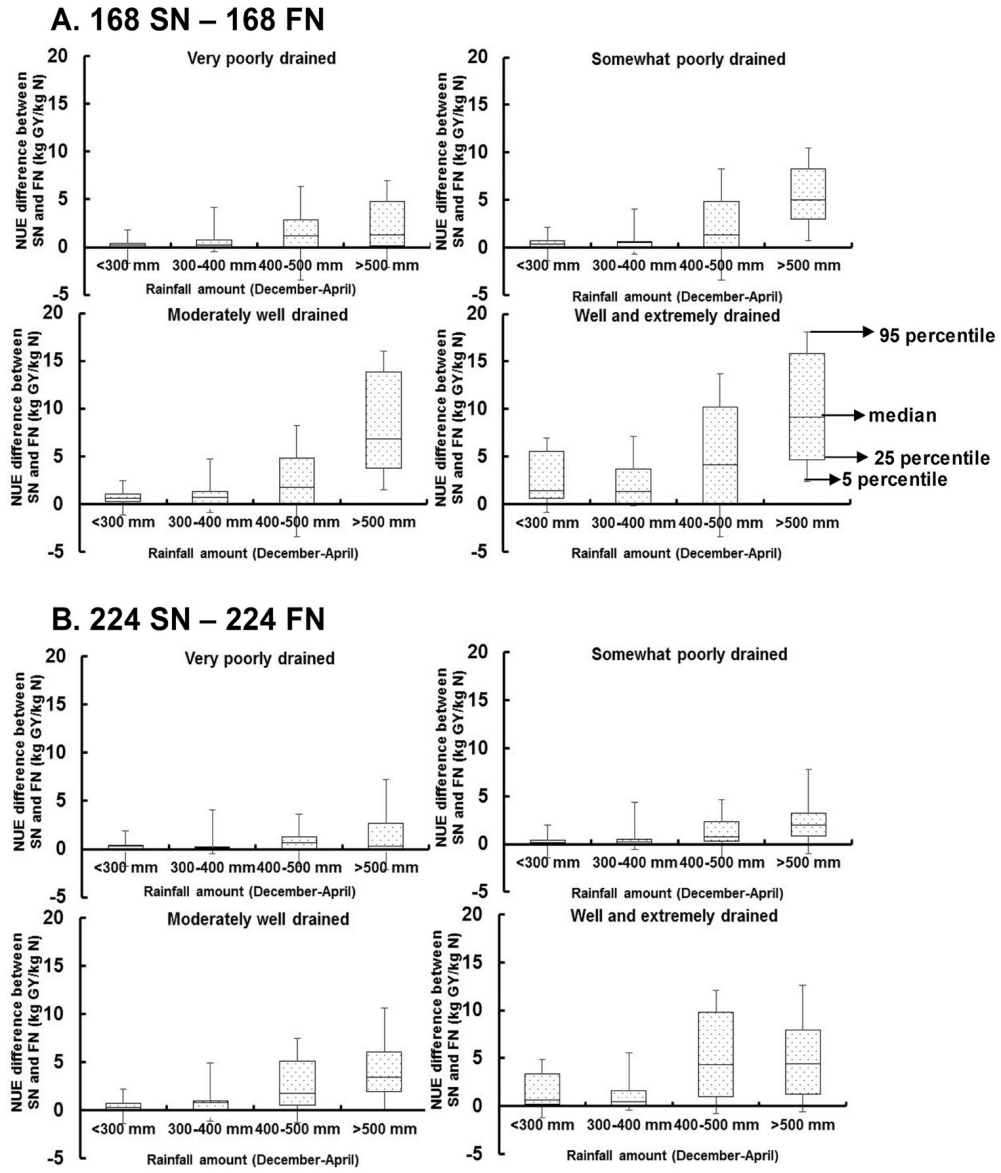
Impacts of cumulative rainfall on N use efficiency. Differences in N use efficiency for spring compared with fall fertilizer N application within each site at two N rates (168 and 224 kg N ha^-1^) as influenced by cumulative early spring rainfall (December through April) and soil drainage class during 2011–2015 in Illinois.

When cumulative winter rainfall was < 300 mm, median NUE differences for SN vs FN among four soil drainage classes ranged 0–1.4 kg GY/kg N and 0–0.6 kg GY/kg N at 168 kg N ha^−1^ and 224 kg N ha^−1^, respectively (Fig 8). However, these differences in NUE increased with increasing cumulative winter rainfall amounts. When cumulative winter rainfall was > 500 mm, the median NUE difference between SN and FN was 1.3–9.2 kg GY/kg N and 0.1–4.4 kg GY/kg N at 168 kg N ha^−1^ and 224 kg N ha^−1^, respectively during 2011–2015. Among the soil drainage classes, the highest difference between SN and FN occurred in well and extremely drained soils (4.4–9.2 kg GY/kg N) and somewhat poorly and moderately well drained soils (2.0–6.7 kg GY/kg N), as compared to poorly and very poorly drained soils (0–1.3 kg GY/kg N).

**Fig 9:**
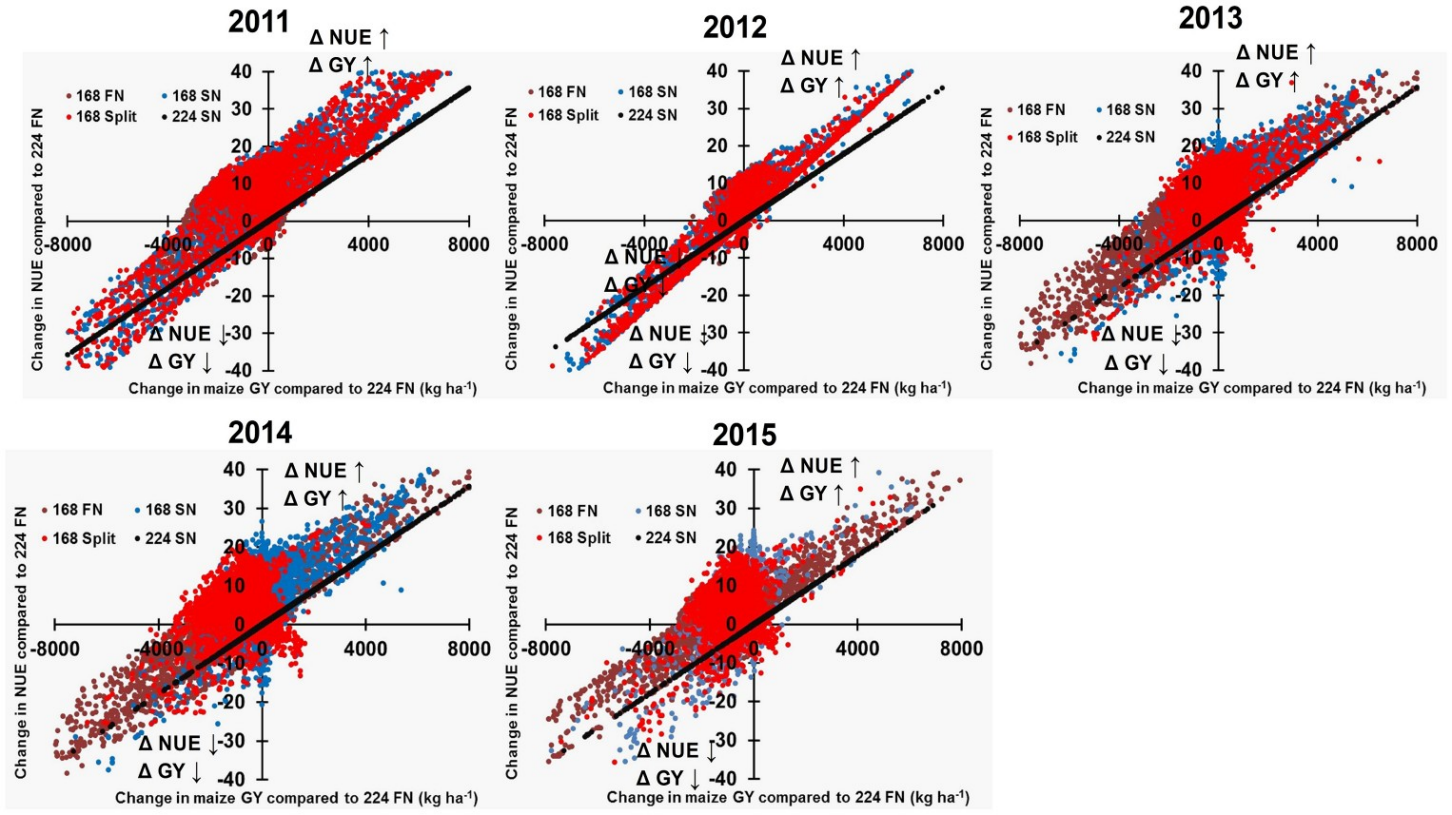
Assessment of simultaneous changes in N use efficiency and grain yield. Changes in N use efficiency (kg GY/kg N) and grain yield (%) for each fertilizer treatment relative to 224 FN (224 kg N ha^-1^ applied in fall) within each site in Illinois during 2011–2015. 224 FN (224 kg N ha^-1^ applied on 1 December); 224 SN (224 kg N ha^-1^ applied on 1 May); 168 FN (168 kg N ha^-1^ applied on 1 December); 168 SN (168 kg N ha^-1^ applied on 1 May); 168 Split (112 kg N ha^-1^ applied on 1 December + 56 kg N ha^-1^ applied on 1 May). The data from southern Illinois watersheds have been excluded as FN is not applied in southern Illinois.

When changes in maize GY and NUE were assessed together, simultaneous increases in maize GY and NUE occurred in 18–40% of cases when comparing 168 FN to 224 FN, and 29–50% of cases when comparing 168 SN and 168 Split to 224 FN during 2011–2015 (Fig 9). In contrast, 224 SN resulted in 53–73% of simulations with simultaneous increases in maize GY and NUE compared to 224 FN during the study period.

## Discussion

To our knowledge, this paper provides the first DSSAT-based evaluation of N application amount and timing effects on maize GY and NUE at the watershed scale in Illinois. In our study, winter rainfall patterns were critical in influencing maize GY as well NUE, and thereby potential N losses to environment. For example, simulations indicated relatively negligible differences in maize GY between 224 SN and FN in 2012, 2014, and 2015 (Figs 5 and 6). However, greater differences in maize GY occurred in 2011 and 2013. Consistent with previous work documenting field-scale N losses, these yield increases were largely driven by cumulative winter rainfall, as 2011 and 2013 received 18–40% greater December through April rainfall (427–490 mm) compared to other years (291–349 mm). In contrast, small yield differences between SN and FN in 2015 were likely associated with this year receiving the lowest winter rainfall (291±143 mm) (Fig 6).

From an environmental standpoint, it is generally accepted that the efficiency of FN is lower than SN, as FN is susceptible to losses for a longer time before plant utilization [13, 14]. However, the impacts of SN compared to FN on maize GY have been variable in the empirical literature. In an earlier comprehensive review, Bundy (1986) determined that SN compared to FN was 10–15% more efficient in increasing maize GY in the U.S. Midwest. In a 4-year field experiment on medium- to fine-textured soils of central and northern Illinois, maize GY was 10% higher with SN than FN at 134 kg N ha^−1^, while no differences in maize GY were observed at 201 kg N ha^−1^ [56]. Using 5 years of field data in Minnesota, Randall & Mulla [21] reported that maize GY was15% greater for SN than FN at 134 kg N ha^-1^, but 5% greater for SN at 202 kg N ha^-1^. Therefore, from an agronomic standpoint our simulations confirm that a portion of FN can be lost to the environment before maize planting in years with greater winter rainfall, which is in agreement with prior field trials (e.g. [19, 13]).

Cumulative winter rainfall was also a dominant factor influencing the effects of fertilizer amount and application timing on NUE (Fig 7). Winter rainfall amounts > 500 mm were found to be an apparent threshold for NUE increasing with SN compared to FN, likely owing to higher N losses through leaching and/or denitrification. For example, the effectiveness of SN in increasing NUE compared to FN was greatest in 2013, since higher winter rainfall occurred in 2013 compared to other years (Fig 4). Dry conditions were also important in our study, with soil moisture limitations in 2012 resulting in NUE values 2-3-fold lower (2.0–8.6 kg GY/kg N) than other years (17.6–26.1kg GY/kg N), such that few differences were observed in GY and NUE between fertilizer amounts and application timing. In general, environmental factors that affect crop growth and/or the capability of soil to supply N such as lack of soil moisture will have the potential to alter NUE [20]. For maize production in South Dakota, Kim et al. [20] reported that irrigation increased NUE due to soil moisture improving N transport from soil to plant roots. High soil moisture can also reduce nitrate sorption in large soil pores, contributing to overall higher N availability for the maize crop [57]. In 13 field experiments in Nebraska, O’Neill et al. [58] reported that NUE was higher in adequate compared to deficit soil moisture regimes.

The simulated median NUE estimates reported here (17.6–26.1 kg GY/kg N) are within the range of other studies on maize production systems of the U.S. Midwest [59, 60]. In irrigated maize cropping systems of Nebraska, NUE ranged from 20–22 kg GY/kg N in continuous maize [59] to 30 kg GY/kg N in maize-soybean systems [60]. Regardless of fertilizer application time in the present study, NUE was greater in the three treatments receiving 168 kg N ha^-1^ than the two treatments with 224 kg N ha^-1^. Apparent NUE is determined by physiological efficiency and N recovery, which are controlled by variety, climate, soil type, and moisture availability [61]. Crop response to N fertilizer inputs generally follows the law of diminishing returns, where incremental gains in N supply are accompanied by relatively lower increase in maize GY due to progressive limitation of other plant growth factors [5, 62, 63]. Field studies have shown that NUE decreases with increasing fertilizer amounts in the U.S. Midwest [56, 59]. In irrigated maize in Nebraska, NUE was 30–38 kg GY/kg N fertilizer at 50 kg N ha^−1^, which decreased to 20–22 kg GY/kg N fertilizer when fertilizer amount was increased to 130 kg N ha^−1^ [59]. From an agronomic standpoint, higher NUE implies relatively greater N uptake per unit of applied fertilizer by crops which makes relatively less N available for leaching, denitrification, and volatilization losses. Consistent with established theory, our simulations indicate NUE decreased at higher N input levels, resulting in a higher fraction of applied N lost to the environment (Fig 7).

To meet ambitious nutrient loss reduction goals in the U.S. Midwest, simultaneous increases in yield and NUE are desired. Federal and state agencies including the Illinois Environmental Protection Agency, the Illinois Department of Agriculture, and a multi-stakeholder Policy Working Group have set an intermediate target of reducing N loads in Illinois by 15% by 2025 from the 1980–1996 baseline [10]. Our results suggest that when N rate is held constant at 224 kg N ha^-1^, switching from FN to SN simultaneously increased maize GY in more than half of simulations each year, ranging from 53–73% during the study period (Fig 9). However, it should be considered that yield gains resulting from SN applications were relatively modest, with only 20% of simulations increasing yields above 3.3% when averaged across years, the majority occurring in 2013 alone. In Illinois, it is estimated that 1.3 million ha of maize receive 100% N fertilizer as FN which is 5–6 months before planting occurs, increasing the potential for environmental N losses [13, 64]. Gentry et al. [65] estimated that delaying N fertilizer application time from FN to SN reduced nitrate loads by 17–20% in two watersheds located in east-central Illinois in 2010. While crop model simulations provide one opportunity to estimate the likelihood of potential yield increases with SN, this information must be incorporated with field trial results (further discussed below) along with management logistics, economic considerations, and additional environmental stewardship goals when making N application timing decisions in this region. Given the current preference for fall field operations in Illinois, the potential yield benefits of SN reported here need to be weighed against the possible risks of encountering wet spring weather. Moreover, it should be considered that decisions related to fall or spring N application timing are likely to become more challenging in the future. It has been estimated that climate change impacts may reduce the number of days suitable for spring field work (Tomasek et al., 2017), further increasing the pressure on spring field operations in Illinois.

**Fig 10:**
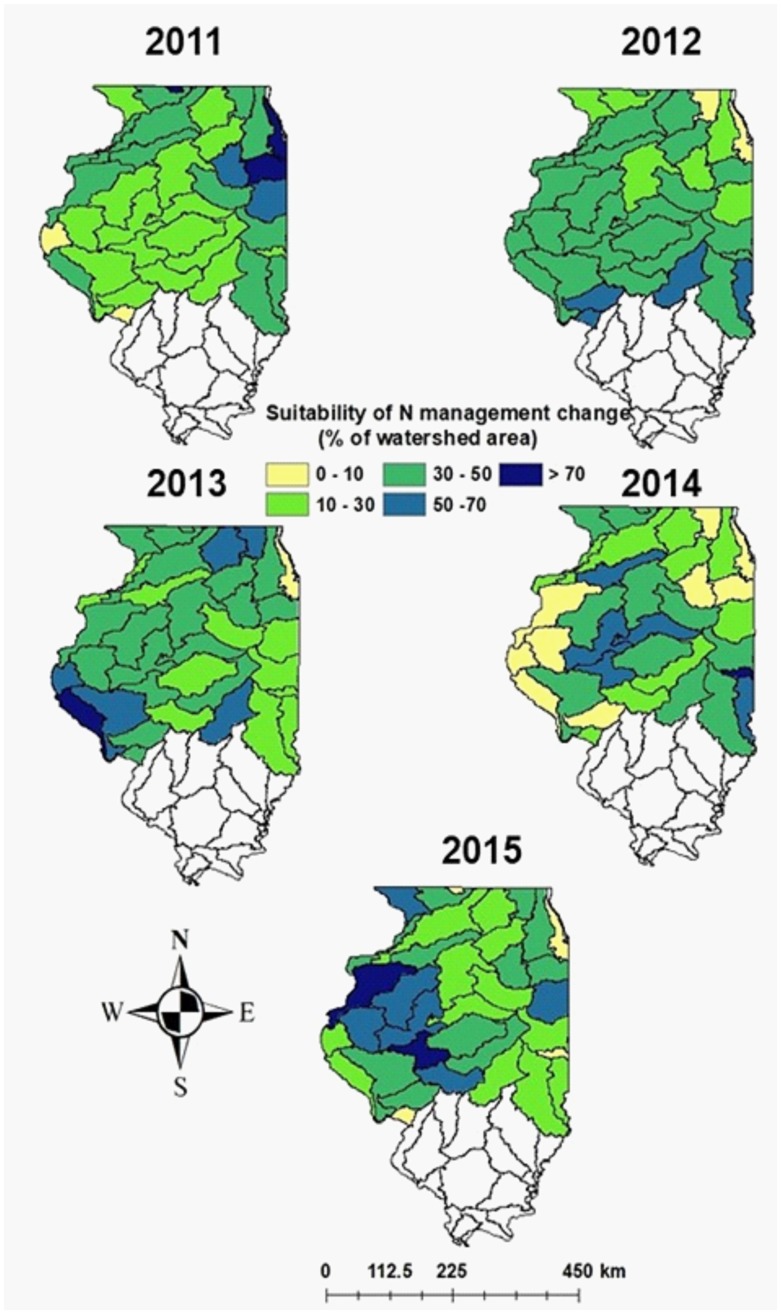
Probability of maize grain yield increase for 224 FN compared to 190 SN. The suitability of the combined practice of reducing N fertilizer amount by 15% (224 to 190 kg N ha^-1^) and delaying application time from fall to spring was based on the percentage of simulations with yield increases greater than 100 kg ha^-1^ within each watershed. The data from southern Illinois watersheds have been excluded as FN is not applied in southern Illinois.

In addition to delaying N fertilizer application timing from fall to spring, a reduction in N rates is generally recommended to decrease N pollution in Illinois [10, 65]. Our simulations revealed that GY and NUE were either simultaneously maintained or increased in only 29–50% of cases when changes in N timing were combined with an N rate reduction of 25% (168 SN and 168 Split) compared to 224 FN for 2011–2015 (Fig 9). Similarly, our simulations show a wide range of impacts on maize GY occurred when switching from FN to SN and reducing N rates by 15% (from 224 FN to 190 SN) across the study period, with the probability of yield increases varying between <10% to >70% of simulation points within a watershed (Fig 10). However, across years only 1–3 watersheds had > 70% probability of a positive GY increase for the 190 SN management scenario. In total, approximately 60% of simulations led to yield gains in 5–37 watersheds when 224 FN was switched to 190 SN (Fig 10), suggesting that yield losses are less likely to occur with a 15 vs. 25% reduction in N rate. Similar to above, an important factor is the consistency of potential yield gains with SN, as the benefits must be attractive enough to outweigh the risks associated with not performing FN application. When assessed on a probability basis within each watershed, 32–36 out of 40 watersheds had <50% probability of yield increases during the study period.

In summary, a contribution of this geospatial assessment is its broad scope, accounting for potential interactions between soil N dynamics and maize GY across a wide range of soil types and climate conditions. However, this study was based on crop model simulations which may not necessarily match field data. Importantly, recent field data in Illinois based replicated on-farm trials do not indicate a clear yield advantage for SN compared to FN, underscoring the need to interpret both field- and model-based results when developing informed decision-making. Another limitation of this work is that the model was not calibrated under the full range of possible conditions in Illinois, highlighting the need for future field experiments to further guide farm-level and policy decisions related to N management. In these efforts it should be considered that to meet nitrate load reduction targets outlined in the ILNLRS (2016), potential yield outcomes due to changes in N management will be more important for some watersheds compared to others. During 2011–2015, our simulations provide evidence that there is a risk that yield losses may occur in approximately <30% to >90% of simulations when N fertilizer rates are reduced by 15% while delaying application time from FN to SN. The results suggest it may be challenging to simultaneously improve maize GY and NUE following N rate reductions of 15 or 25% in this region. Additional field experiments across a wider variety of crop management practices and soil types are warranted, particularly to identify the extent to which potential yield tradeoffs may be associated with N rate reductions. If a similar risk of yield losses related to changes in N application amount and timing are observed, it is likely that greater emphasis should be placed on other approaches such as cover crops and edge-of-field practices (e.g. bioreactors, wetlands, and buffers) to meet N load reduction targets in Illinois [10].

## Supporting information

S1 Fig Automation procedure used to run the calibrated DSSAT model at 3042 points in Illinois each year. (DOCX)

S2 Fig Average maize planting dates in crop districts of Illinois during 2011–2015 based on weekly USDA National Agricultural Statistics Service Illinois crop progress and condition reports. (DOCX)

S1 Table Description of six N tracking experimental sites in Illinois used in DSSAT calibration. (DOCX)

S2 Table Description of the three N rate experimental sites in Illinois used in DSSAT calibration. (DOCX)

S3 Table Description of fifteen experiments in Illinois used in DSSAT validation. (DOCX)

S1 Data Underlying data. Files contain simulation results for each treatment across the study years.
(ZIP)
