# Supplementary material for: Simulating nitrogen management impacts on maize production in the U.S. Midwest
Source: PLoS One. 2018 Oct 22;13(10):e0201825. doi: 10.1371/journal.pone.0201825 (PMC6197644; doi:10.1371/journal.pone.0201825)

**S1 Fig.** Automation procedure used to run the calibrated DSSAT model at 3042 points in Illinois each year.


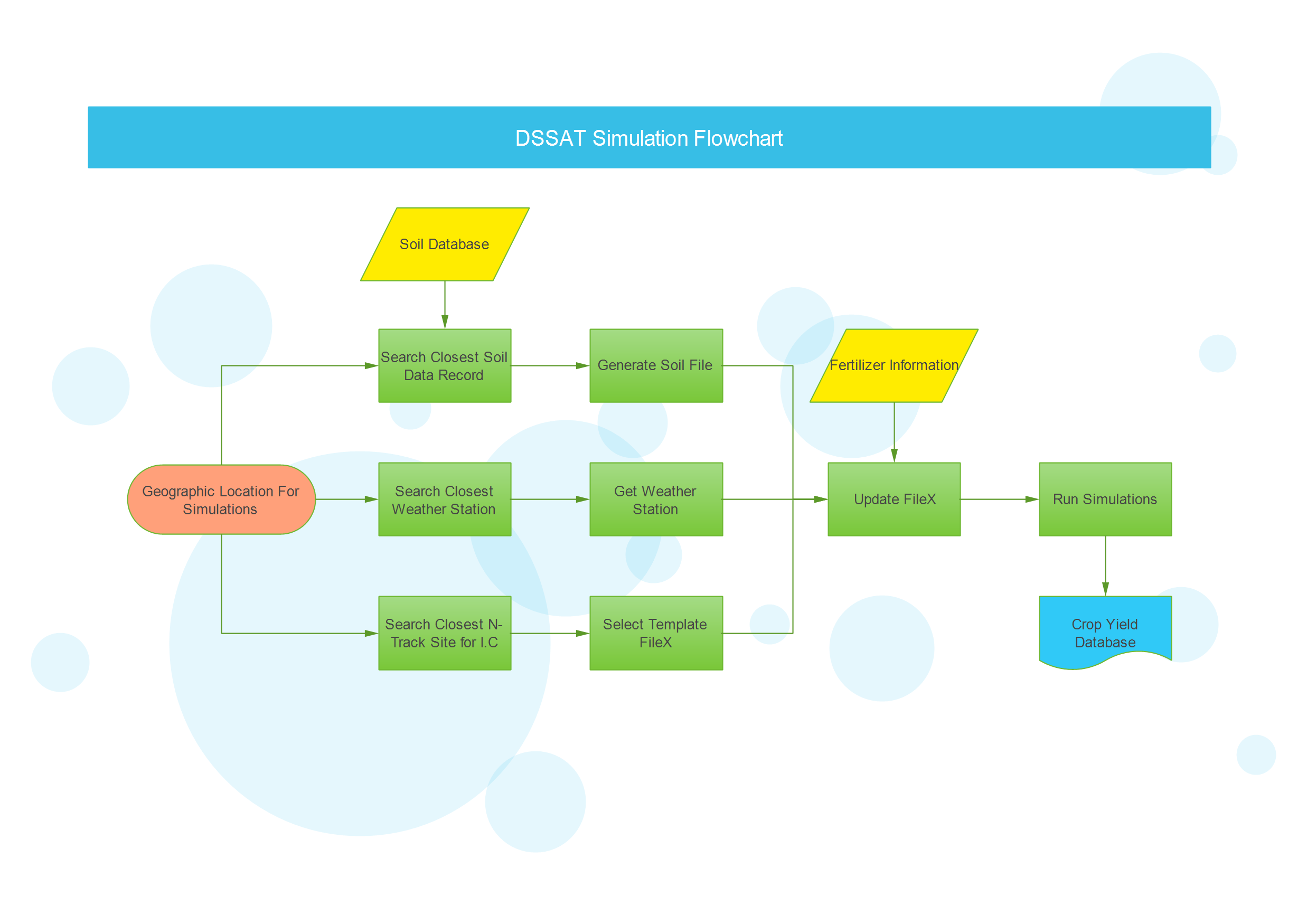

Supplement: S1 Fig — (DOCX) [file pone.0201825.s001.docx]
