# Supplementary material for: Simulating nitrogen management impacts on maize production in the U.S. Midwest
Source: PLoS One. 2018 Oct 22;13(10):e0201825. doi: 10.1371/journal.pone.0201825 (PMC6197644; doi:10.1371/journal.pone.0201825)

**S2 Fig**

Average maize planting dates in crop districts of Illinois during 2011–2015 based on weekly USDA National Agricultural Statistics Service’s Illinois crop progress and condition reports.


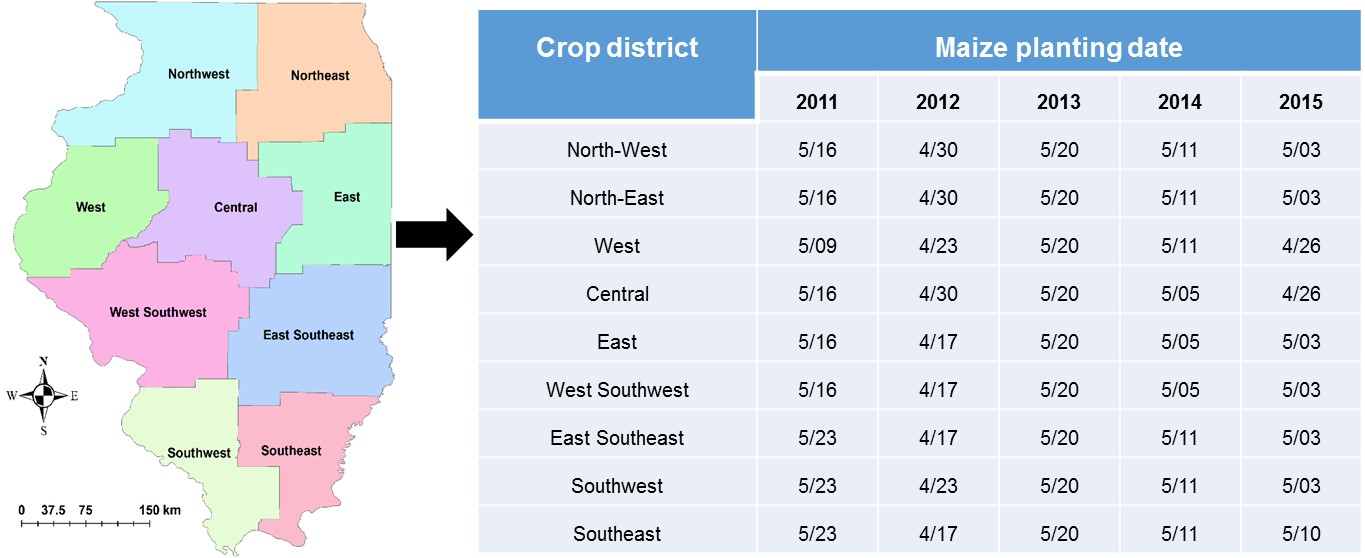

Supplement: S2 Fig — (DOCX) [file pone.0201825.s002.docx]
