# Supplementary material for: Simulating nitrogen management impacts on maize production in the U.S. Midwest
Source: PLoS One. 2018 Oct 22;13(10):e0201825. doi: 10.1371/journal.pone.0201825 (PMC6197644; doi:10.1371/journal.pone.0201825)
Supplement: S1 Table — (DOCX) [file pone.0201825.s003.docx]

**S1 Table**

Description of six N tracking experimental sites in Illinois used in DSSAT calibration. In northern and central Illinois, fertilizer treatments included: 1) control, 2) 224-Fall (224 kg N ha^-1^ applied as anhydrous ammonia in fall), 3) 224-Spring (224 kg N ha^-1^ applied as anhydrous ammonia in spring), 4) 224-Split1 (56 kg N ha^-1^ applied as UAN at planting + 168 kg N ha^-1^ applied as UAN at V5 stage), 5) 224-Split2 (112 kg N ha^-1^ applied as anhydrous ammonia in fall + 56 kg N ha^-1^ applied as UAN at planting and again at V5 stage). In southern Illinois, fertilizer treatments included: 1) control, 2) 168-Spring (168 kg N ha^-1^ applied as UAN at planting), 3) 168-Split1 (56 kg N ha^-1^ applied as UAN at planting + 112 kg N ha^-1^ applied as UAN at V5 stage), and 4) 168-Split2 (56 kg N ha^-1^ applied as UAN at planting + 112 kg N ha^-1^ applied as UAN at V9 stage).

| Experimental site | Latitude | Longitude | Cultivar | Planting | Anthesis | Physiological maturity | Soil ammonium  (mg [N]/ kg soil) | | Soil nitrate  (mg [N]/ kg soil) | |
| --- | --- | --- | --- | --- | --- | --- | --- | --- | --- | --- |
|  |  |  |  |  | –––––– Days after planting ––––– | | 0–30 cm | 30–60 cm | 0–30 cm | 30–60 cm |
| –––––––––––––––––––––––– Northern and central Illinois –––––––––––––––––––––––– | | | | | | | | | | |
| DeKalb | 41.93 | 88.75 | P0636 | 05/01 | 82 | 146 | 5.4 | 4.1 | 5.2 | 2.1 |
| Monmouth | 40.91 | 90.65 | P1221 | 04/21 | 86 | 148 | 4.6 | 3.4 | 2.5 | 1.5 |
| Urbana | 40.11 | 88.21 | DKC 62-08 | 04/23 | 79 | – | 2.3 | 1.8 | 7.4 | 3.0 |
| Perry | 39.78 | 90.75 | FA 61SX1 | 04/22 | 79 | – | 2.3 | 3.8 | 6.4 | 2.9 |
| –––––––––––––––––––––––– Southern Illinois –––––––––––––––––––––––– | | | | | | | | | | |
| Brownstown | 39.00 | 88.95 | P1151 | 05/03 | 67 | 120 | 5.2 | 4.3 | 5.4 | 1.9 |
| Dixon Springs | 37.46 | 88.72 | P1498 | 05/07 | 63 | 112 | 2.9 | 3.8 | 10.0 | 3.9 |
