# Supplementary material for: Simulating nitrogen management impacts on maize production in the U.S. Midwest
Source: PLoS One. 2018 Oct 22;13(10):e0201825. doi: 10.1371/journal.pone.0201825 (PMC6197644; doi:10.1371/journal.pone.0201825)
Supplement: S2 Table — (DOCX) [file pone.0201825.s004.docx]

**S2 Table**

Location and planting date for the three N rate experimental sites in Illinois used in DSSAT calibration. The following N rate treatments were included at each site: 0, 56, 112, 168, 224, and 280 kg N ha^-1^.

| Experiment | Latitude | Longitude | Planting |
| --- | --- | --- | --- |
| ––––––2014–––––– | | | |
| Site 1 | 40.08 | –88.226 | 05/05 |
| Site 2 | 41.843 | –88.855 | 05/11 |
| Site 3 | 40.926 | –90.725 | 05/11 |
| ––––––2015–––––– | | | |
| Site 1 | 40.05 | –88.231 | 05/03 |
| Site 2 | 41.848 | –88.866 | 05/03 |
| Site 3 | 40.926 | –90.726 | 05/03 |
