# Supplementary material for: Simulating nitrogen management impacts on maize production in the U.S. Midwest
Source: PLoS One. 2018 Oct 22;13(10):e0201825. doi: 10.1371/journal.pone.0201825 (PMC6197644; doi:10.1371/journal.pone.0201825)
Supplement: S3 Table — (DOCX) [file pone.0201825.s005.docx]

**S3 Table**

Soil drainage rate and soil organic carbon for fifteen experiments in Illinois used in DSSAT validation.

| Site | Latitude | Longitude | Soil drainage rate | Soil organic carbon |
| --- | --- | --- | --- | --- |
| –––––––– Nitrogen rate trials –––––––– | | | | |
| Site 1 | 38.955 | -88.960 | 0.25 | 0.8 |
| Site 2 | 41.841 | -88.859 | 0.40 | 1.8 |
| Site 3 | 37.457 | -88.718 | 0.60 | 0.7 |
| Site 4 | 40.927 | -90.725 | 0.25 | 1.0 |
| Site 5 | 39.800 | -90.820 | 0.25 | 0.2 |
| Site 6 | 40.048 | -88.229 | 0.40 | 1.8 |
| –––––––– FN vs SN application time –––––––– | | | | |
| Site 1 | 39.952 | -88.566 | 0.05 | 2.3 |
| Site 2 | 39.567 | -89.854 | 0.05 | 1.2 |
| –––––––– FN vs split application time –––––––– | | | | |
| Site 1 | 39.992 | -88.632 | 0.60 | 0.9 |
| Site 2 | 39.831 | -89.340 | 0.05 | 2.6 |
| Site 3 | 39.970 | -88.316 | 0.60 | 1.7 |
| Site 4 | 39.969 | -88.468 | 0.25 | 2.2 |
| –––––––– SN vs split application time –––––––– | | | | |
| Site 1 | 40.5898 | -88.0768 | 0.25 | 1.9 |
| Site 2 | 39.6223 | -90.1372 | 0.60 | 0.8 |
| Site 3 | 39.6057 | -90.4437 | 0.40 | 0.6 |

FN: fall applied N; SN: Spring applied N.
